# Rare Case of Rickettsiosis Caused by *Rickettsia monacensis*, Portugal, 2021 

**DOI:** 10.3201/eid2805.211836

**Published:** 2022-05

**Authors:** Rita de Sousa, Marta Leal dos Santos, Claudina Cruz, Vasco Almeida, Ana Raquel Garrote, Freddy Ramirez, Diana Seixas, Maria J. Manata, Fernando Maltez

**Affiliations:** National Institute of Health Dr. Ricardo Jorge, Águas de Moura, Portugal (R. de Sousa);; Hospital de Curry Cabral, Lisbon, Portugal (M. Leal dos Santos, C. Cruz, V. Almeida, A.R. Garrote, F. Ramirez, D. Seixas, M.J. Manata, F. Maltez)

**Keywords:** Rickettsia monacensis, tick-borne disease, eschar, rash, rickettsiosis, molecular detection, Ixodes ricinus, ticks, IrITA2, IrITA3, IRS3, IRS4,IrR/Munich, Portugal, rickettsia, vector-borne infections, bacteria

## Abstract

We report a case of rickettsiosis caused by *Rickettsia monacensis* in an immunocompetent 67-year-old man in Portugal who had eschar, erythematous rash, and an attached *Ixodes ricinus* tick. Seroconversion and eschar biopsy led to confirmed diagnosis by PCR. Physicians should be aware of this rare rickettsiosis, especially in geographic regions with the vector.

*Rickettsia monacensis,* spotted fever group rickettsiae (SFGR), are bacteria transmitted by *Ixodes* spp. ticks and are rarely reported as causing disease in humans. Few cases have been documented and laboratory confirmed (*1*–*4*). *R. monacensis* infection causing Mediterranean spotted fever (MSF)–like rickettsiosis was described in 2007 for 2 patients from La Rioja and the Basque Country, Spain, followed by 1 case in Italy (2012) and 2 cases in South Korea (2017 and 2019) (*1**–**4*). Despite the few human infections described, *R. monacensis* is frequently found (0.5%– 42.5%) in *Ixodes ricinus* ticks in Europe, including Portugal and North Africa, and in another *Ixodes* species tick in Asia (*3*–*5*).

Three previously reported rickettsioses in Portugal were MSF caused by *R. conorii*, tickborne lymphadenopathy caused by *R. slovaca*, and lymphangitis-associated rickettsiosis caused by *R. sibirica mongolitimonae* (*6*–*8*). We report *R. monacensis* infection in a human and *Rickettsia* in the attached tick.

In February 2021, a 67-year-old man with alcoholism–associated dilated cardiomyopathy and diabetes mellitus type 2 was hospitalized in Lisbon, Portugal. The patient reported a 5-day history of fever and appearance of rash on day 3 of fever onset. He lived in Lisbon and had traveled to a rural area 5 days before symptom onset. At admission, he had fever, fatigue, myalgia, and anorexia. Physical examination showed disperse upper-body erythematous exanthema, palmo-plantar erythema, and an eschar surrounded by erythema on his upper left back (Figure). An engorged female *I. ricinus* tick was removed from the patient. Laboratory evaluation showed hematologic, hepatic, and renal abnormalities; anemia (hemoglobin 9.7 g/dL); lymphopenia (420 cells/μL); thrombocytopenia (38,000 platelets/mm^3^); and increased serum levels of creatinine (2.23 mg/dL), alanine aminotransferase (73 IU/L), aspartate aminotransferase (89 IU/L), creatine phosphokinase (116 IU/dL), lactate dehydrogenase (148 IU/L), and C-reactive protein (159.5 mg/L). Electrocardiography findings were unremarkable. Oral doxycycline (200 mg/d) was empirically started on hospitalization day 1. 

**Figure:**
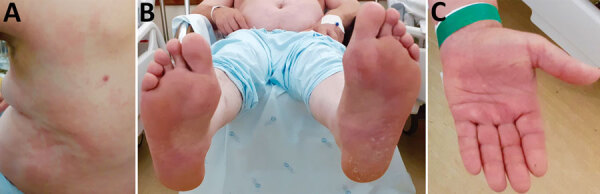
Patient with rickettsiosis caused by *Rickettsia monacensis*, Portugal, 2021. A) Rash and eschar; B) rash on soles; C) rash on palms.

After the patient had been hospitalized for 12 hours and received 1 dose of doxycycline, we biopsied the eschar and collected a blood sample. PCR and DNA sequence analysis of partial fragments of *omp*A and *glt*A genes from the tick and biopsy samples showed 100% identity with nucleotide sequences of *R. monacensis* (GenBank accession no. LN794217). Screening for *Borrelia* DNA in the tick was negative.

For antibody testing we used an immunofluorescence assay from FOCUS Diagnostics (https://www.focusdx.com), which used commercial *R. conorii* IFA substrate slides for IgG and IgM; results demonstrated seroconversion within 2 weeks in consecutively collected samples. We detected no antibodies in the acute-phase serum sample collected on day 6 after symptom onset, and we detected reactive antibodies against SFGR (IgM titer 32, IgG titer 128) in the second sample only, collected 3 weeks after illness onset (*9*). Supplemental methods and results are in the Appendix.

After 48 hours of antimicrobial therapy, the patient was afebrile; after 4 days, exanthema was completely resolved; and after 7 days, all symptoms had resolved. The patient was discharged and scheduled for outpatient follow-up. 

We confirm that *R. monacensis* caused disease in this patient. Very few cases of human infection with *R. monacensis* have been reported, possibly because this species is not considered to be very pathogenic and for most patients might cause self-limited infection (*1*–*5*). Another hypothesis is that cases have been misdiagnosed or confirmed by serology only, which cannot distinguish among SFGR species (*8*,*9*). Moreover, if cases occur in the autumn/winter, when adult *I. ricinus* ticks are more active and outside the peak season (June–September) for MSF, some physicians might not think of rickettsiosis as the cause, particularly if there is no epidemiologic context and clinical findings are not highly suggestive. 

For the patient reported here, we identified an eschar, as was done for the 3 other patients from Italy and South Korea (Table). However, the first 2 patients identified in Spain did not have any sign of an eschar. We are unaware whether any specific patient host factors could be associated with *R. monacensis* infection, but alcoholism in the patient reported here could have been a risk factor for severity (*8*). With exception of the patient from Italy, all patients were >59 years of age, including the patient from Portugal, and at least 3 were hospitalized. In general, it would seem that older persons are more susceptible to disease, even when infected with low-pathogenicity *Rickettsia*. For instance, in the case report of an 8-year-old child from Croatia with Lyme borreliosis, in whom *R. monacensis* DNA was also detected in a skin biopsy of the erythema migrans tissue, antibodies against *Borrelia* were detected but not antibodies against SFGR (*10*).

**Table:** Clinical features and laboratory diagnosis of patients with *Rickettsia monacensis* infection, 2003–2021

Feature	Patient 1, La Rioja, Spain (*1*)	Patient 2, Basque, Spain (*1*)	Patient 3, Sardinia, Italy (*2*)	Patient 4, South Korea (*3**)*	Patient 5, South Korea (*4*)	Patient 6, Portugal (this study)
Epidemiologic						
Age, y/ sex	84/M	59/F	28/M	73/M	75/F	67/M
Date of onset	Jun 2003	Sep 2003	Apr 2011	2006	Oct 2019	Feb 2021
Tick bite history	NK	Yes	NK	NK	NK	Yes
Clinical						
Fever, ºC (ºF)	Yes, 39.5º (103.1º)	Yes, 38º (100.4º)	Yes, 38.2º (100.8º)	Yes, 40º (104.0º)	Yes, 38.4º (101.1º)	Yes, 39.9º (103.8º)
Eschar (location)	No	No	Yes (calf)	Yes (back)	Yes (scalp)	Yes (back)
Rash	Yes	Yes	No	Yes	Yes	Yes
Type	Maculopapular	Erythematous		Maculopapular	Maculopapular	Erythematous
Including palm and soles	Yes					Yes
Headache	Yes	Yes	Yes	Yes	NK	Yes
Lymphadenopathy	NK	NK	NK	Yes	Yes	NK
Laboratory test results						
SFGR IFA titer						
Sample 1	<40 IgG	2,560 IgG	128 IgG	320 (IgM + IgG)	<16 IgM/32 IgG, negative	Negative
Sample 2	1,280 IgG (26 wk later)	1,280 IgG	NA	NA	16 IgM/128 IgG (2 mo)	32 IgM /128 IgG (2 wk)
Culture, blood/biopsy	Positive	Negative	NA	Positive	NA	NA
PCR detection, blood and/or skin biopsy	Positive	Positive	Positive	NK	Positive	Positive
Co-infections with other pathogens	NK	NK	NK	NK	*Orientia tsutsugamushi*	NK
Treatment						
Hospitalization	NA	NA	Yes	Yes	Yes	Yes
Antimicrobial drug	Doxycycline 100 mg every 12 h for 10 d	Doxycycline 100 mg every 12 h for 10 d	Doxycycline 100 mg every 12 h for 7 d	Azithromycin 500 mg, 1 dose	Doxycycline 200 mg/d	Doxycycline 200 mg/d


This case of infection with *R. monacensis*, formerly considered to be of low pathogenicity and found in *Ixodes* spp. ticks, was associated with disease in an immunocompetent patient. Other cases may be underdiagnosed, particularly outside the usual summer months when MSF cases peak in Portugal. Moreover, because *R. monacensis* shares the same vector as *Borrelia* spp. and these co-infections have been detected, physicians should be aware of this rickettsiosis, especially in areas where the vector is present.

Appendix Supplementary methods and results for study of rare case of rickettsiosis caused by *Rickettsia monacensis*, Portugal, 2021.
