# Supplementary material for: Rare Case of Rickettsiosis Caused by Rickettsia monacensis, Portugal, 2021
Source: Emerg Infect Dis. 2022 May;28(5):1068–71. doi: 10.3201/eid2805.211836 (PMC9045422; doi:10.3201/eid2805.211836)
Supplement: Appendix — Supplementary methods and results for study of rare case of rickettsiosis caused by Rickettsia monacensis, Portugal, 2021. [file 21-1836-Techapp-s1.pdf]

Article DOI: https://doi.org/10.3201/eid2805.211836

Rare Case of Rickettsiosis Caused by *Rickettsia monacensis*, Portugal, 2021

Appendix

Methods

DNA was extracted from skin biopsy and tick using a MagCore HF 16 Plus automated nucleic acid extractor (RBC Bioscience, Taiwan) and MagCore® Genomic DNA tissue kit.

For tick molecular identification a conventional PCR targeting partial region of mitochondrial marker 16S ribosomal RNA gene using the primer set 16S+1/16S-1, which amplifies approximately 456 bp, was performed as previously described by Black & Piesman (*1*).

PCR targeting the rickettsial genes for citrate synthase (*gltA*) and outer membrane protein A (*ompA*) were performed with specific primers. For the amplification of skin biopsy DNA we have used a nested-PCR using the primer sets RpCS.415/ RpCS.1220 and RpCS.877/RpCS.1258n to amplify partial fragment of citrate synthase (*gltA*) gene and primer sets Rr190.70p/Rr190.701n and Rr190.70p/190.602n to amplify a fragment of *omp*A gene, according previously described by Portillo and collaborators (*2*). For tick DNA amplification we have used regular conventional PCR using the RpCS.415/ RpCS.1220 and Rr190.70p/190.602n primer sets of *glt*A and *omp*A genes (*2*).

PCR were performed in a 50-µL reaction mixture containing 25 µL of the High Fidelity PCR Master Kit buffer (Roche Diagnostics, GmbH, Mannheim, Germany), 2 µL of each primer at 0.2 µmol/L, and 10 µL genomic DNA. Amplification was performed in a DNA thermocycler (T-3 thermoblock T, Biometra, Goettingen, Germany) according previous described amplification protocols (*2*). For each reaction, a negative control (water) and a positive control of *R. rickettsii* were included. Five microliters of the PCR products were resolved by electrophoresis in 1.2% agarose gel with GelRed® Nucleic Acid Gel Stain (Biotium, California,

USA) and examined by UV transillumination. PCR products of the expected size were purified with ExoSAP-IT™ PCR Product Cleanup Reagent and sequenced in an ABI automated sequencer (Applied Biosystems, Foster City, CA, USA) by using the ABI PRISM Big Dye Terminator Cycle Sequencing Ready Reaction Kit (Applied Biosystems), according to the protocols supplied by manufacturers. All sequences were determined by the consensus of the forward and reverse sequence analysis. The sequences of the *gltA*, *ompA*, amplicons were aligned with the corresponding sequences of other *Rickettsia* species available in GenBank/EMBL database, by using BLASTN software.

We also have extract the DNA from the tick and did PCR for the tick mitochondrial marker 16S ribosomal RNA gene using the primer set of Black and Piesman (*1*) to confirm the *Ixodes ricinus* species. We have add the GenBank accessing number of our sequence (OK484994).

Results

The sequence from the skin biopsy designated PoHuR34655 exhibited nucleotide sequence of *gltA* (341/341 bp) and *omp*A (488/488bp) 100% identical to *Rickettsia monacensis* IrMunich strain (LN 794217). The tick sequence designated PoTiR20 was also 100% (766/766 bp) identical to *glt*A of *Rickettsia monacensis* IrMunich strain (LN 794217). For *omp*A the sequence of the tick is identical and have the same length (bp) to the sequence of the biopsy specimen. The sequence which identified the tick as *Ixodes ricinus* was designated PTtick 20.

Nucleotide Sequence Accession Numbers

The GenBank nucleotide sequence accession numbers for partial sequences of *gltA*, *ompA*, genes generated in this study for PoHuR34655 are: OK504620, OK504619, respectively; gltA partial sequence for PoTiR20 is OK504621. GenBank accessing number for PTtick20 : OK484994 (*I.ricinus* 16S rDNA).

References

1. Black WC IV, Piesman J. Phylogeny of hard- and soft-tick taxa (Acari: Ixodida) based on mitochondrial 16S rDNA sequences. Proc Natl Acad Sci U S A. 1994;91:10034–8. PubMed https://doi.org/10.1073/pnas.91.21.10034

2. Portillo A, de Sousa R, Santibáñez S, Duarte A, Edouard S, Fonseca IP, et al. Guidelines for the detection of *Rickettsia* spp. Vector Borne Zoonotic Dis. 2017;17:23–32. https://doi.org/10.1089/vbz.2016.1966
